# A low-dimensional representation of arm movements and hand grip forces in post-stroke individuals

**DOI:** 10.1038/s41598-022-11806-4

**Published:** 2022-05-09

**Authors:** Christoph M. Kanzler, Giuseppe Averta, Anne Schwarz, Jeremia P. O. Held, Roger Gassert, Antonio Bicchi, Marco Santello, Olivier Lambercy, Matteo Bianchi

**Affiliations:** 1 Department of Health Sciences and Technology, Rehabilitation Engineering Laboratory, Institute of Robotics and Intelligent Systems, ETH Zurich, Zurich, Switzerland; 2 Future Health Technologies, Singapore-ETH Centre, Campus for Research Excellence and Technological Enterprise (CREATE), Singapore, Singapore; 3 Dipartimento di Ingegneria dell’Informazione, Research Center “Enrico Piaggio”, University of Pisa, Pisa, Italy; 4 Soft Robotics for Human Cooperation and Rehabilitation, Fondazione Istituto Italiano di Tecnologia, Genova, Italy; 5 Department of Neurology, Vascular Neurology and Neurorehabilitation, University Hospital and University of Zurich, Zurich, Switzerland; 6 Biomedical Signals and Systems (BSS), University of Twente, Enschede, The Netherlands; 7 School of Biological and Health Systems Engineering, Arizona State University, Tempe, AZ USA

**Keywords:** Diseases of the nervous system, Motor control, Sensorimotor processing

## Abstract

Characterizing post-stroke impairments in the sensorimotor control of arm and hand is essential to better understand altered mechanisms of movement generation. Herein, we used a decomposition algorithm to characterize impairments in end-effector velocity and hand grip force data collected from an instrumented functional task in 83 healthy control and 27 chronic post-stroke individuals with mild-to-moderate impairments. According to kinematic and kinetic raw data, post-stroke individuals showed reduced functional performance during all task phases. After applying the decomposition algorithm, we observed that the behavioural data from healthy controls relies on a low-dimensional representation and demonstrated that this representation is mostly preserved post-stroke. Further, it emerged that reduced functional performance post-stroke correlates to an abnormal variance distribution of the behavioural representation, except when reducing hand grip forces. This suggests that the behavioural repertoire in these post-stroke individuals is mostly preserved, thereby pointing towards therapeutic strategies that optimize movement quality and the reduction of grip forces to improve performance of daily life activities post-stroke.

## Introduction

Impairments in the sensorimotor control of arm and hand are observed in up to 77% persons after stroke and strongly affects persons independence and quality of life^1^. Providing a comprehensive characterization of these impairments is of fundamental importance for advancing the mechanistic understanding of altered movement composition in neurological disorders, as well as for informing the design of effective and patient-tailored neurorehabilitation approaches^2^.

In research studies, these impairments have usually been assessed by relying on validated and standardized clinical tests, such as the Fugl-Meyer assessment for the upper extremity (FMA-UE) or the Action Research Arm Test (ARAT)^2–4^. These scales are typically based on a coarse and subjective description of movement quality, or on the time to complete functional tasks. Consequently, such assessments are not able to quantitatively describe movement and grip force patterns, even though those are fundamental to better capture the control of goal-directed actions by the central nervous system (CNS) and stroke-induced alterations therein^5^.

Technology-based assessments have been introduced with the aim of providing a more fine-grained characterization of impairments in upper limb control^6–10^. These approaches allow to record behavioural time-series data, such as kinematics and kinetics, and enable to precisely study the impaired control of arm movements and hand grip forces during functional tasks. Traditionally, these time-series have been analyzed using discrete metrics that capture specific landmarks, for example, the maximum velocity or the number of velocity peaks, or the frequency distribution of the signal, for example, the spectral arc length^6,11,12^. Thus, this type of analysis does not allow to maintain information about the temporal evolution and adaptation of motor control in goal-directed tasks, which is vital to fully understand how person after stroke perform daily life activities. To better capture these aspects, so-called decomposition algorithms can instead be applied to the recorded time-series^13,14^. One of such algorithms is functional principal component analysis (fPCA), a statistical method that decomposes time-series into a low-dimensional representation of orthogonal basis functions that can have a subject-specific shape and are hierarchically ordered based on the variance of the input signal that they explain^15,16^. Crucially, such a low-dimensional representation is expected to inform on the architecture that underpins the observed behaviour^14,15^. For example, the application of such decomposition algorithms to electromyographic data acquired during goal-directed arm movements allowed to unveil a low-dimensional neural control architecture that was found to be preserved in post-stroke patients with mild to moderate upper limb impairments^17–20^. Despite their relevance for daily life activities, considerably less emphasis has been placed on using decomposition algorithms to study post-stroke impairments in the control of endpoint kinematics and hand grip forces during goal-directed movements^21–26^.

**Figure 1:**
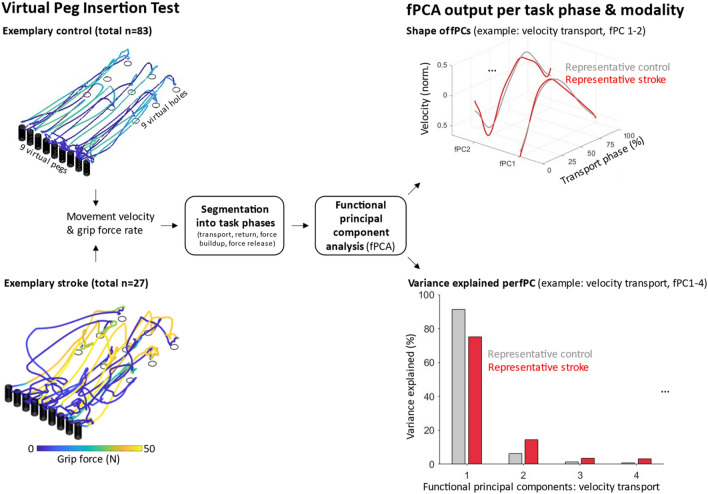
Overview of the approach to capture the control of arm movements and grip forces. Kinematic and kinetic time-series were collected in a control and post-stroke population using a technology-based assessment with a goal-directed functional task, the Virtual Peg Insertion Test (representative raw data in left panel). After a pre-processing and temporal segmentation (middle panel), functional Principal Component Analysis (fPCA) was applied. fPCA allows reconstructing the time-series with a set of low dimensional basis functions, named fPCs. These fPCs can have a subject-specific shape and explain certain variance of the input signal, which was compared between a representative control and post-stroke individual, indicating a similar shape of the fPCs and a different distribution of variance explained between the subjects (right panel). The control was male and 40 years old. The post-stroke individual presented a mild upper limb sensorimotor impairment according to the Fugl-Meyer assessment for the upper extremity (score 55, age 52 years, male).

In this work, we aim to provide a detailed characterization of post-stroke impairments in the control of endpoint kinematics and hand grip forces through the usage of a decomposition algorithm, namely fPCA. For this purpose, we relied on endpoint velocity (kinematics) and grip force rate (kinetics) data from chronic post-stroke individuals and age-matched healthy controls during a goal-directed technology-based assessment, the Virtual Peg Insertion Test (VPIT, Figs. 1, SM1)^12,27,28^. These data were processed and temporally segmented into four task phases that focused either on the simultaneous control of arm movements and grip forces (*transport, return*), or more on isolated grip force control (*force buildup, force release*; details in *Methods*). To avoid a potential influence of different durations of these phases on the kinematic and kinetic behaviour, the time-series were temporally rescaled using dynamic time warping^15^. Subsequently, the aligned time-series were processed using fPCA to identify an optimal basis of fPCs encoding the original data. Then, the shape of the initial time-series and the fPCs, as well as the variance explained by each fPC were statistically compared between control and post-stroke conditions.

Building upon previous research on the control of arm movements^17,18^ and the literature on grip force control^21,22^, we hypothesized that (i) post-stroke impairments are visible in the control of arm movements and grip forces as described by kinematic and kinetic data, (ii) that these data can be accurately reconstructed in a low-dimensional space that is similar between controls and post-stroke individuals, and that (iii) post-stroke impairments instead affect the distribution of variance in the low-dimensional space.

## Results

VPIT data used for the analysis were obtained from 27 post-stroke individuals (14 female, time post-stroke 89.1 [49.1, 147.7] weeks, reported as median [25th-percentile, 75th-percentile], details in Table SM1), with 23 and 27 of them successfully completing the VPIT protocol (i.e., they can insert all nine pegs in all five task repetitions) with the paretic and non-paretic body side, respectively. This implies that four participants were not able to complete the task with the paretic side, which resulted from too severe upper limb impairments (FMA-UE scores of these four participants were 28, 38, 38, and 44). The age of the all participants was 59.0 [53.5, 68.5] years with a mild to moderate level of sensorimotor impairment (paretic side: FMA-UE 49 [41, 57]) and activity limitations (paretic side: ARAT, 47 [39, 55]). In addition, 83 healthy controls (41 female, age 60.2 [50.3, 70.9] years) were selected as age-matched control group from a normative database^12^.

During the experiment, the VPIT recorded kinematic information, specifically the endpoint position, and kinetic information, such as the grip forces applied to the handle (Figs. 1 and SM1). From these data, we derived the endpoint velocity and the grip force rate profiles for our analysis based on the focus of previous research^21,29^. These data were temporally segmented to identify four task phases that were defined in prior work and have specific physiological requirements^12^: peg transport (ballistic movement between peg pick-up and insertion into a hole; active grip force modulation required), peg return (ballistic movement between the insertion of a peg and the next peg pick-up; grip force modulation not required), force buildup (phase of maximal grip force production) and force release (phase of maximal grip force reduction). Subsequently, fPCA was applied separately on all phases for the kinematic and kinetic signals, resulting in six independent conditions (peg transport and peg return for the velocity and all the four phases for the grip force rate signals). Afterwards, the low-dimensional representation of the behavioural signals was described using the shape and variance explained by each fPC.

### Shape and consistency of the fPCs

**Figure 2:**
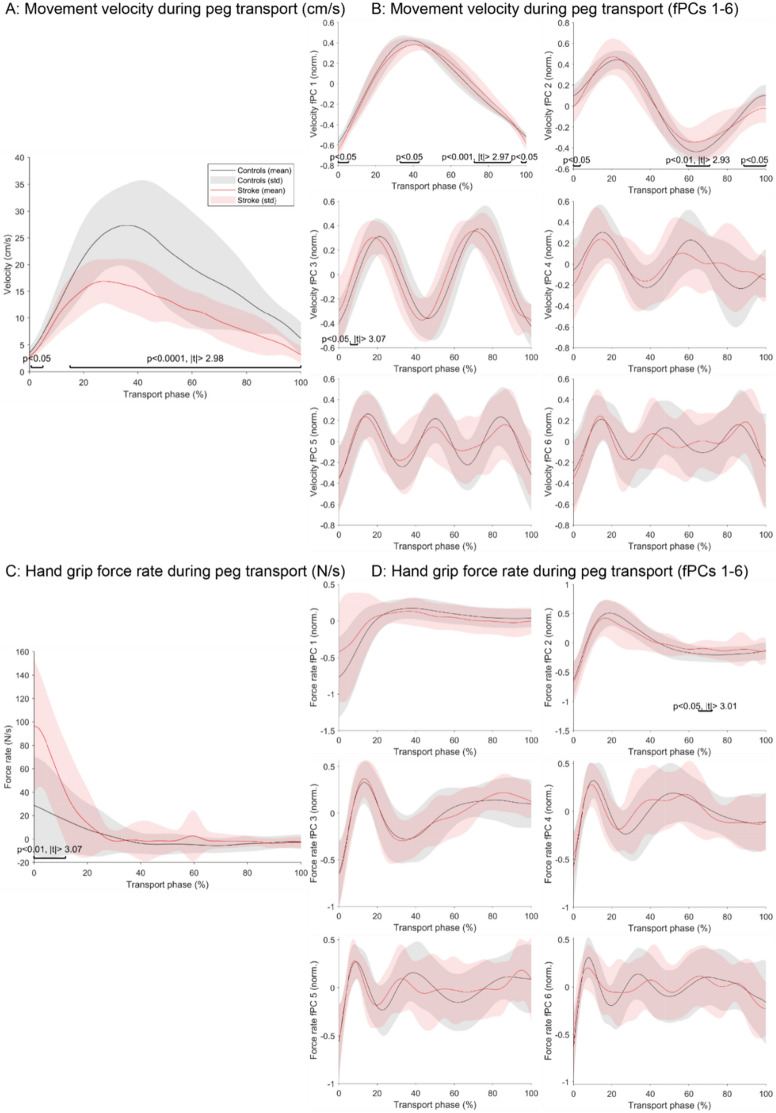
Movement and hand grip force coordination during the transport phase of the functional task. The preprocessed end-effector velocity (**A**) and grip force rate (**C**) signals were visualized for the paretic side of post-stroke subjects (red) and a healthy age-matched control population (gray). In addition, the shapes of the fPCs for the velocity (**B**) and the force rate (**D**) signals were visualized. The time-series of the stroke and control population were compared using statistical parametric mapping and the p- and t-values of significant periods annotated.

Regarding the kinematic aspects (endpoint velocity) of the peg transport, it is evident that the raw data (Fig. 2A) as well as the first fPC were bell-shaped whereas higher order fPCs had sinusoidal-like shape (Fig. 2B). In the shape of raw velocity profiles, clear and continuous significant differences between the control and post-stroke population could be observed (*P* < 0.05 and *P* < 0.0001, |*t*|> 2.98, Cohen’s *d* = 1.59). In the shape of the fPCs, only few periods with slight, significant differences between the populations could be observed in fPC 1–3.

To quantify the similarity of the fPC shape across participants, their variability was evaluated using the average interquartile range (IQR) along the trajectory. We observed a strong coherence across control participants for the first (IQR = 0.06) and second fPC (IQR = 0.08) for the velocity transport, while higher variability (IQR ≥ 0.24) was observed for higher order fPCs. In the post-stroke population, the fPCs were coherent across participants, although this is characterized by a greater variability (IQR = 0.14 and 0.16 for the first and the second fPC respectively; IQR ≥ 0.28 for higher order fPCs).

In the kinetic domain (grip force rate) during the peg transport phase, the shape of the raw data and the first fPC indicates an initial adaptation of the force followed by a convergence towards zero (Fig. 2C and D). In the shape of the raw grip force rate profiles, clear significant differences between the control and post-stroke population could be observed in the beginning of the task phase (*P* < 0.01, |t|> 3.07, *d* = − 1.37). In the shape of the fPCs, only a brief period in the second fPC showed a slight, significant difference (*P* < 0.05, |t|> 3.01, *d* = − 0.76).

The inter-subject variability (IQR) of the first and second fPC in the control population was 0.11 and 0.15, respectively, whereas higher order components showed increased inter-subject variability (IQR ≥ 0.32). In the post-stroke population, the inter-subject variability of the first and second fPCs was 0.30 and 0.20, respectively.

**Figure 3:**
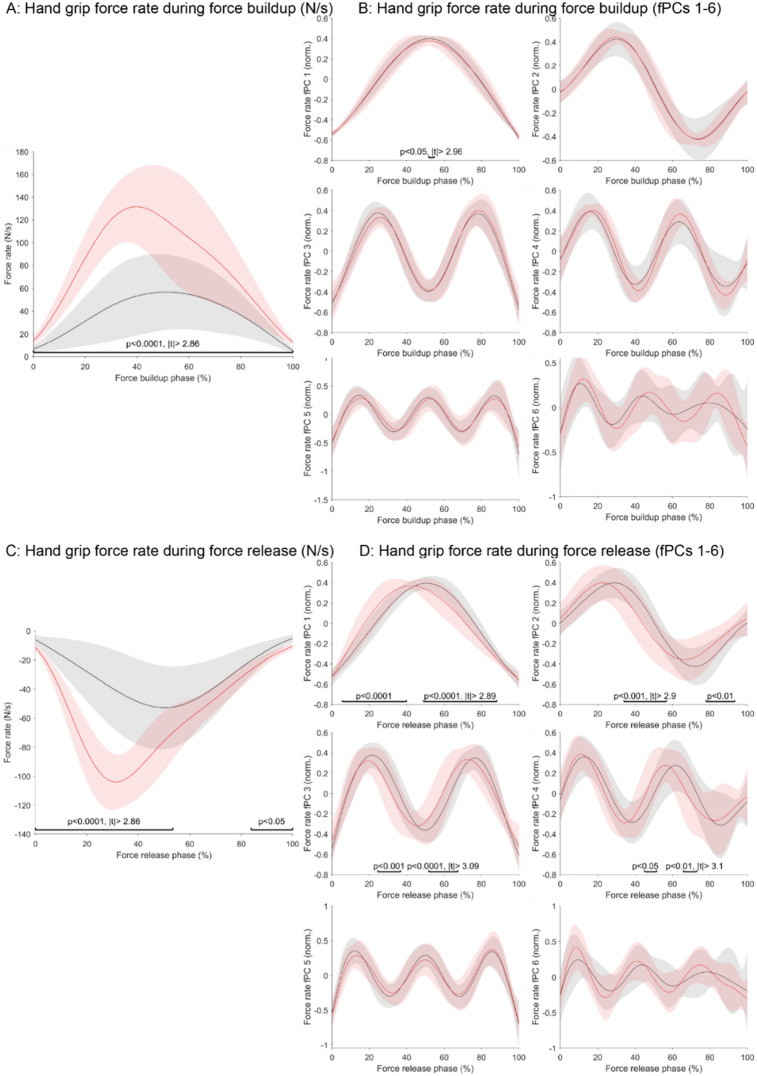
Grip force coordination during the force buildup and the force release phases of the functional task. The preprocessed end-effector grip force rate signals during buildup (**A**) and release (**C**) phases were visualized for the paretic side of post-stroke subjects (red) and a healthy age-matched control population (gray). In addition, the shapes of the fPCs for the buildup (**B**) and the release (**D**) signals were visualized. The time-series of the stroke and control population were compared using statistical parametric mapping and the *P*- and t-values of significant periods annotated.

Additionally, the same analysis was applied to the *force buildup* (Fig. 3A and B) *and force release phases* (Fig. 3C and D), which focus on the periods of maximal force production and reduction that occurred during the handling of each peg, respectively. With that, these phases focus more specifically on grip force control. For both phases, a clear bell-shaped force rate profile could be observed in the raw data and strong, significant, and continuous alterations were present in post-stroke compared to control participants (force buildup: *P* < 0.0001, |t|> 2.86, *d* = − 2.15; force release: *P* < 0.001 and *P* < 0.05, |t|> 2.86, *d* = 2.14). Also, the first fPC of the force rate buildup exhibited a clear congruent bell-shape across both populations, and significant differences between the populations were only found for one short period across all fPCs. Furthermore, for control participants, the IQR was 0.08 for the first fPC and 0.10 for the second functional PC of the force buildup. For stroke participants, the IQR was 0.15 and 0.14 for the first and the second functional PCs, respectively.

For the first fPC of the force rate release, the bell-shape of the post-stroke diverged from the one of the control population, with considerable significant differences emerging for the first (*P* < 0.0001 and *P* < 0.0001, |t|> 2.89, *d* = 1.02), second (*P* < 0.001 and *P* < 0.01, |t|> 2.9, *d* = 1.03), third (*P* < 0.001 and *P* < 0.0001, |t|> 3.09, *d* = − 0.85), and fourth (*P* < 0.05 and *P* < 0.01, |t|> 3.1, *d* = 0.84) fPC. Lastly, the inter-subject variability was 0.10 and 0.12 for the first and second fPC of control participants, whereas it was 0.15 and 0.20 for the first and second fPC of post-stroke participants, respectively.

The same analysis was carried out for the return phase and the results are reported in the supplementary material (Fig. SM2).

### Variance explained by fPCs

**Figure 4:**
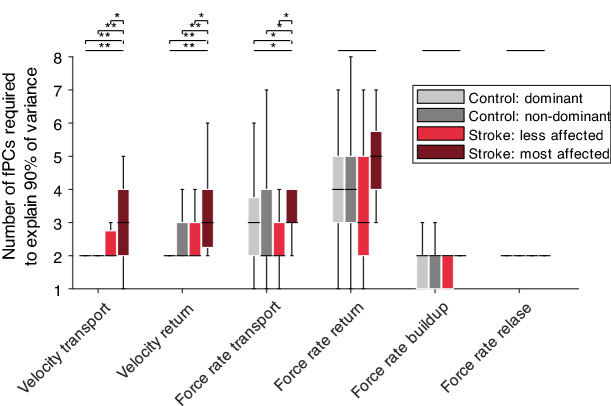
Number of fPCs required to explain 90% of the variance in the input signal. This information was provided for each task phase and modality (end-effector velocity and grip force rate), visualized for the paretic side of post-stroke subjects (dark red), the non-paretic side of post-stroke subjects (light red), the dominant arm of the healthy age-matched control population (light gray) and the non-dominant arm of the healthy age-matched control population (dark gray). Horizontal black line indicates median, boxes represent the IQR, and the whiskers the min and max value within 1.5IQR. Horizontal solid line on top indicates results from an omnibus test and dashed lines from post-hoc tests. *Indicates *P* < 0.05, **P < 0.001.

Beyond the analysis of the peculiar shapes that the fPCs show across conditions and across tasks, it is interesting to also analyse the amount of variance explained by these components. For the control population, it is relevant to note that more than 90% of the total variance of the kinematic data can be explained by just two fPCs, while three components are required for the kinetic domain during transport and return (Fig. 4). More specifically, the number of fPCs required to accurately reconstruct (i.e. to explain at least 90% of the total variance) the trajectories was 2.0 ± 0.0 for the velocity transport/return of the dominant side of controls (Fig. 4), and significantly increased for the paretic side of post-stroke individuals (velocity transport 3 ± 2, *t* = − 52.3, *P* < 0.0001; velocity return: 3.0 ± 1.8, *t* = − 68.8, *P* < 0.0001). Similar trends were observed for the force rate transport (control dominant 3 ± 1.8, stroke paretic 3 ± 1, *t* = − 39.4, *P* = 0.0243), but not for the remaining analyses focusing on grip forces (omnibus-tests *P* > 0.05). This slight differentiation between the transport and return phase is because both phases are not identical, as during the transport phase a grip force of 2 N needs to be applied to transport a peg, whereas during the return phase no specific force needs to be applied to the handle, which results in rather high inter-participant variability.

**Figure 5:**
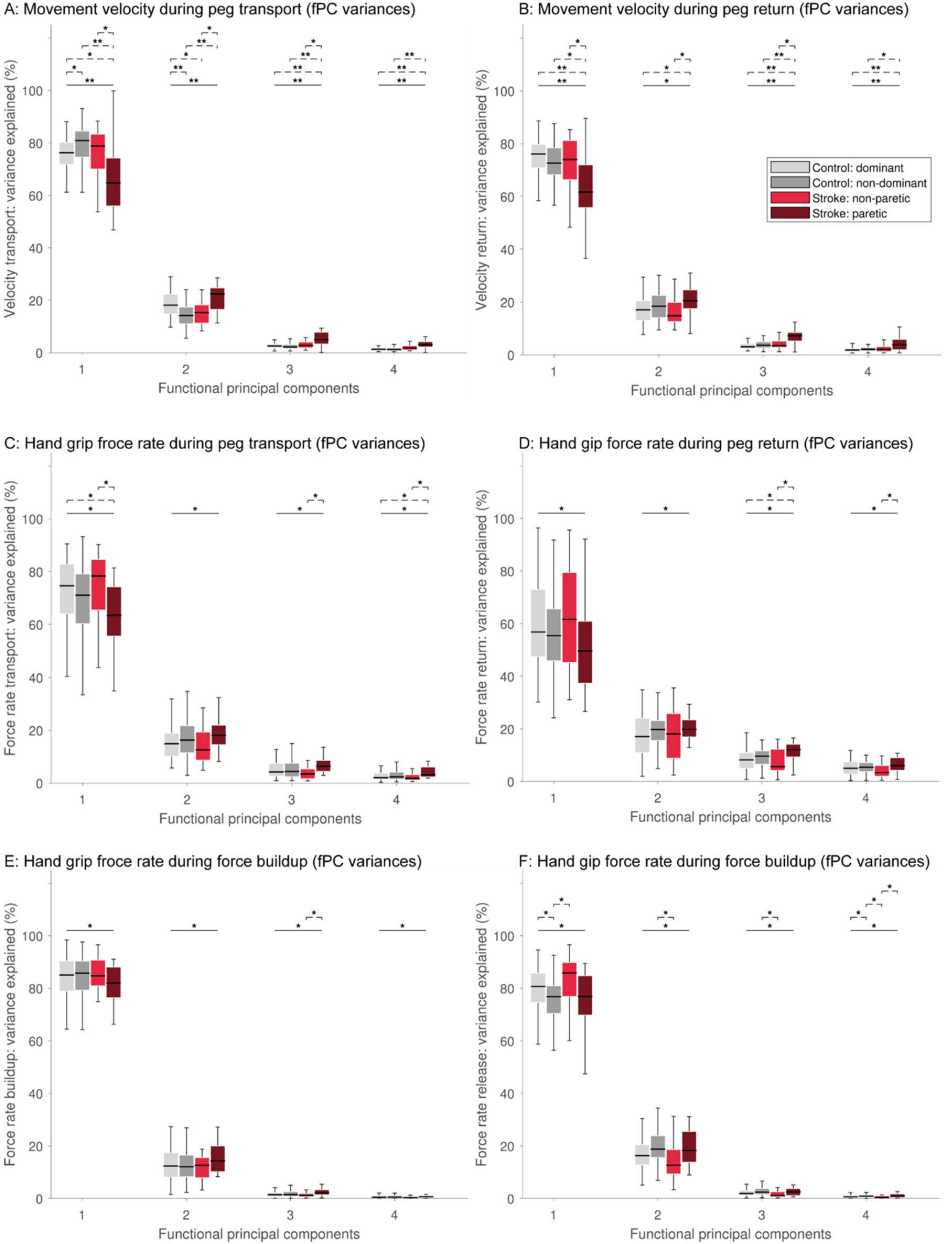
Population-level comparison of variance explained per fPC, task phase, and modality. Horizontal solid line indicates results from an omnibus test and dashed lines from post-hoc tests. *Indicates *P* < 0.05, **P < 0.001.

Figure 5 provides a more detailed analysis on the variability explained by the fPCs for each phase in control and post-stroke participants. In the following, the statistical results are reported for the transport velocity (Fig. 5A) for representative example data. Therein, the first fPC accounted for 76.2% ± 8.5% of the variance in control individuals. Clear significant differences in the variance explained by the first fPC could be observed between control individuals and the paretic body side of post-stroke participants (64.7%± 18.2%, t = 50.0, *P* = 0.0038), as well as between the non-paretic (68.% ± 13.3%) and paretic side of post-stroke participants (t = 61.5, *P* = 0.0030). On the contrary, the second to fourth fPCs of the velocity transport signal explained significantly more variance in the paretic side of post-stroke individuals than controls (e.g., fPC 2 controls non-dominant 14.1% ± 6.4%, post-stroke paretic 22.4% ± 8.2, t = − 69.6, *P* < 0.0001).

### Correlation with conventional clinical scales

The variance explained per fPC was correlated with conventional clinical scales (Tables 1 and SM2). For this purpose, the FMA-UE was used as an established clinical measure of upper limb sensorimotor impairments. Across all signals and task phases, the Spearman correlation was 0.42 ± 0.17 for the first fPC, − 0.31 ± 0.15 for the second fPC, − 0.46 ± 0.16 for the third fPC, and − 0.44 ±0.18 for the fourth fPC (all results in Table 1). All correlations were statistically significant except for fPC 2 of force rate return, fPC 1, 2, 4, 5, 6 for force rate during the force buildup phase, and fPC 3 and 4 for force rate during the force release phase. For completeness, the correlation analysis was also implemented for the ARAT, which yielded similar results as for the FMA-UE (details in Table SM2).

**Table 1:** Correlations between the variance explained per fPC and the FMA-UE.

Signal and task phase	Correlations of fPCs with FMA-UE					
	fPC1	fPC2	fPC3	fPC4	fPC5	fPC6
Velocity transport	0.63**	− 0.51**	− 0.58**	− 0.62**	− 0.64**	− 0.64**
Force rate transport	0.52**	− 0.37*	− 0.54**	− 0.59**	− 0.62**	− 0.60**
Velocity return	0.58**	− 0.36*	− 0.69**	− 0.55**	− 0.54**	− 0.49**
Force rate return	0.30*	− 0.09	− 0.33*	− 0.41*	− 0.29	− 0.33*
Force rate buildup	0.22	− 0.17	− 0.36*	− 0.19	− 0.16	− 0.26
Force rate release	0.31*	− 0.34*	− 0.28	− 0.29	− 0.38*	− 0.41*

The Spearman correlation analysis was performed for the paretic of post-stroke subjects. Bold indicates significant entries. *P < 0.05. **P < 0.001.

## Discussion

Over the last decades, technology-based assessments and novel metrics describing movement quality greatly advanced the understanding of impaired upper limb sensorimotor control after stroke^6–10,30–33^. However, the use of decomposition algorithms to characterize the behavioural structure of upper limb kinematics and kinetics and its temporal evolution during functional goal-directed tasks has received less attention. This is surprising, given that such approaches previously enabled novel insights into the neural control of electromyographic signals^17,18^ and that such knowledge could help to inform rehabilitation strategies that attempt to improve performance during daily life activities. This work aims to characterize post-stroke impairments in the control of goal-directed arm movements and grip forces post-stroke using a decomposition algorithm, namely fPCA. This technique decomposes time-series into a linear, weighted low-dimensional combination of basis functions (fPCs). By analyzing the variance explained by the fPCs, as well as their shape, we expect to gain novel insights in the behavioural composition of goal-directed movements and grip forces^15,16^. Therefore, fPCA was applied to end-effector velocity and grip force rate data recorded during different phases (transport, return, force buildup, force release) of a goal-directed pick and place task that was performed by 83 controls and 27 chronic post-stroke individuals with mild to moderate sensorimotor impairments^12,27^.

### A low-dimensional representation of arm movements and grip forces

The results presented in this work highlight three important observations: (i) the well-coordinated control of arm movements and grip forces post-stroke is consistently impaired across task phases, thereby confirming our first hypothesis; (ii) the simultaneous control of arm movements and grip forces rely on a low-dimensional representation of fPCs that is mostly preserved post-stroke, thereby partially confirming our second hypothesis; (iii) the reduced functional performance caused by a stroke can be described by a pathological variance distribution of these components, except when coordinating the reduction of grip forces, thereby partially confirming our third hypothesis.

In the following, we discuss these results and their implications in more details. Specifically, we start with the raw data and fPCA-transformed data in control participants and subsequently focus on analyzing how these are affected by pathological alterations. Similarly, we first focus on the case of movement kinematics, followed by the coordination of movement kinematics and grip force kinetics, and conclude with the control of grip forces.

### Control of arm movements

Considering the endpoint velocity during task phases requiring gross movements with (transport phase) and without (return phase) active grip force modulation in the control population, we found that a low-dimensional representation of two fPCs allows to accurately reconstruct the behavioural data. As expected, we found that most of the variance is associated to the first fPC, which is associated to the well-known bell-shaped velocity profile and is also clearly present in the untransformed raw data^29^. In addition, we herein report a second fPC with a distinct sinusoidal-like shape (Fig. 3B), which is not obviously perceivable when inspecting the raw data. Keeping in mind that fPCs are associated with a positive or negative weighting factor, this implies that the second fPC can be interpreted as a means to slow-down the first part of the movement while speeding-up the second part, and, vice versa, speeding-up the first part of the motion while slowing-down the second part. The first two fPCs are strongly consistent across participants, thus supporting the idea that they are indeed key components that describe coordinated goal-directed movements in healthy conditions. In contrast, higher order fPCs seem to be associated with variability during the movement and superimposed with subject-specific local adaptations to the task, as indicated by the significantly lower similarity between higher order components across participants.

Extending the analysis to the post-stroke population, we observed a clear reduction in movement speed and a degradation of the bell-shape profile in the raw data, which is in line with previous studies and expected to relate to a reduced ability to generate and transmit neural commands^34^. Intriguingly, we found a strong coherence in the shape of all fPCs with the corresponding ones of the control population, with only short periods in the fPCs showing alterations. Again, similar to the control population, a consistently higher inter-subject variability characterized higher order components post-stroke. The major difference in terms of fPCA between the control and post-stroke population is in the variability explained by the fPCs. Indeed, the first fPC accounts for a significantly lower percentage of variance in the post-stroke case and, instead, higher order fPCs are required to account for an equivalent amount of variance as in controls. However, our results demonstrate that the shape of all fPCs, including the bell-shaped and the sinusoidal-like one, are preserved post-stroke. In other words, this suggests that the same fPCs are present in controls and post-stroke individuals, and that the reduced functional performance in post-stroke individuals can be described by a suboptimal, pathological variance distribution of these fPCs. Consistent with this interpretation, we observed that decreased variance explained by the bell-shaped component correlated positively with the FMA-UE as an established measure of upper limb impairments, whereas increased variance explained by higher order fPCs correlated negatively with the FMA-UE. This suggests that the abnormal variance distribution of these components describes abnormal task performance post-stroke. Previous work already suggested that movements of post-stroke individuals still rely on the same movement components as in healthy controls, namely bell-shaped sub-movements, but that the spatio-temporal arrangement of these sub-movements is altered post-stroke^30,31^. Herein, we extend these observations by showing that this altered movement arrangement can be explained by few fPCs with an intuitive task-related interpretation.

### Control of arm movements and hand grip forces

The strong dimensionality reduction observed in the kinematic space appears to be paralleled in the kinetic domain in unaffected participants. Considering the transport phase, the first fPC covered 75% of total variability, while the first three fPCs account for over 95% (Fig. 4). Compared to the kinematic domain, the two main fPCs in the kinetic domain show slightly larger inter-subject variability. The role of the first fPC is to decrease the grip force in approximately the first quarter of the gross movement (Fig. 2C,D), while keeping the grip force almost constant during the remaining part of the movement. This observation highlights that participants apply more force than required (2 N) when picking up a virtual peg and rapidly reduce the level of applied forces to achieve resource-efficient task performance. This confirms previous studies that reported a safety margin in the applied forces when initially lifting and transporting objects^22,35,36^. The second kinetic fPC during transport decreases the grip force rate in the initial part of the movement and increases it subsequently. It is interesting to observe that grip forces are mostly adapted during the first quarter of the movement, when most of limb acceleration usually occurs, in line with previous studies^37^. These studies also proposed the concept that movement and grip force are governed through two separate controllers and that the movement controller is able to send control signals to the grip force controller to achieve an online adaption of grip forces^37^. The observation that velocity and grip force rate both seemed to most rapidly change in the beginning of the goal-directed movement (Fig. 2A,C) would support these propositions, as it is likely that rapid communication between the controllers is required to establish such well-coordinated behaviour. Such a concept would also explain the higher variability of the first kinetic basic function compared to the kinematic one, as the kinematic behaviour is used to further fine-tune and adapt the kinetic one.

In post-stroke participants, a clear, significant increase in the grip force rate was observed in the beginning of the transport phase (Fig. 2C). This might be reflective of an abnormally increased safety margin when picking up the virtual object, resulting from impaired somatosensory perception, processing, and integration that is reported in certain post-stroke individuals and can affect the grip force controller^22,37,38^. An additional, non-mutually exclusive, explanation for these observations would be that the task-dependent adaptation of grip forces, as conceptualized by the communication between the movement and grip force controllers, can also be impaired in post-stroke participants. Despite these clear stroke-related alterations in the kinetic raw data during gross movements, it is intriguing that the shape of the fPCs remains similar to the ones of controls (e.g., Fig. 3C,D). Interestingly, in the beginning of the movement, the inter-subject variability of the first kinetic fPC was slightly, but on a population-level non significantly, greater in post-stroke individuals compared to controls. This might suggest that a subpopulation of post-stroke participants has different impairment mechanisms, which could potentially relate to a differential influence of stroke on the grip force controller or on the communication between movement and grip force controller. However, idiosyncratic differences, such as precise lesion location and extent, might also contribute to differences between subpopulations. Further, we observed that the first kinetic fPC during peg transport explained significantly less variance compared to the unaffected controls. Overall, this suggests that on a population level, analogous to the kinematic case, the same components are describing kinetic behaviour during goal-directed movements in controls and post-stroke individuals, but that the variance distribution of these is abnormal. This was also confirmed by the positive correlation of the variance explained by the first kinetic fPC with the FMA-UE and the negative correlation of the variance explained by the other fPCs with the FMA-UE. Through these observations, we provide substantial knowledge on the behavioural components describing movement kinematics and grip force control during functional tasks in post-stroke participants. Clearly, such a detailed description of upper limb behaviour could not have been obtained through functional clinical tasks, such as the FMA-UE or Action Research Arm Test.

### Control of hand grip forces

The phases of maximal increase (force buildup) and decrease (force release) of hand grip forces are independent of a specific phase in the functional task (e.g., transport or return) and allow a more isolated evaluation of grip force control. During these phases, we observed a characteristic bell-shaped component for the force rate in the raw data and as first fPC for the control population, consistent with previous findings^36^. Also, an additional component of sinusoidal-like shape was required to explain 90% of variance (Fig. SM2). Given that the shape of these force rate profiles strongly resembles the one in the kinematic domain during the feedforward-controlled ballistic movement, it is likely that these rapid phases of force control are also governed in a feedforward fashion^22^.

In post-stroke individuals, we observed a clear, abnormal increase in the magnitude of grip force changes (i.e., raw data) for both the force buildup and release phases. Intriguingly, the shape of the fPCs was similar between control and post-stroke participants for the force buildup, but not the release phases (Fig. 3). This suggests that the behavioural components controlling the reduction of grip forces are altered post-stroke, whereas the representation underlying the increase of grip forces is similar to healthy controls. These findings only become evident when inspecting both raw data and transformed fPCs. In general, abnormalities in the increase of grip forces (force buildup) have been associated to abnormal finger flexor activity^22^, whereas abnormalities in the reduction of grip forces (force release) are related to an impaired modulation in the tone of finger flexor muscle^39^. In addition, participants might try to support the reduction of grip forces through slight finger extension. However, this has been shown to trigger involuntary finger flexion due to muscle co-contractions, thus potentially contributing to the observed abnormal behaviour during the reduction of grip forces^40^. Such abnormal finger control has been attributed to the involuntary co-activation of multiple muscle groups as a result of cortical remapping, increased reliance on brainstem pathways with extensively branched projections (e.g., reticulospinal tract), changes in spinal cord excitability, and slight alterations in the mechanical properties of the muscles^40–42^. While somatosensory deficits can affect grip force control as well^22^, these are likely not the primary contributors given that the rapid phases of force buildup and release are expected to underly feedforward control. The observed preservation of the behavioural representation during the increase, but not the release of hand grip forces, in post-stroke individuals suggests that a differential influence of the mentioned neural mechanisms exists for these behaviours. These findings warrant to more precisely study and pinpoint the contribution of these mechanisms in grip force control during functional tasks post-stroke.

### Relation of kinematic and kinetic components to previously identified muscular components

Compared to previous work analyzing the neural control of arm movements using electromyography data^13,17,18^, we herein instead focus on the behavioural level as described by endpoint kinematics and hand grip forces obtained during a functional task. The reason for this is twofold: First, recent research emphasized the importance of studying not only the control of muscular activation patterns, but instead also the control of functional task performance^43,44^, as understanding the latter might be necessary to detect sensorimotor impairments with direct relevance for daily life, which could serve as targets for therapeutic interventions. Second, compared to electromyographic systems, end-effector based approaches, such as the VPIT, are more likely to be integrated as outcome measures for clinical studies because of their greater usability, as demonstrated by the rapid time to set up the patient and administer the assessment (median of 16.6 min with the paretic body side) in persons with mild to moderate sensorimotor impairments^28^. Obtaining a comprehensive assessment of movement kinematics and kinetics in this subpopulation of post-stroke individuals is of clinical interest, because it could guide the stratification into one of the interventions readily available for this population, for example constrained induced movement therapy or high intensity therapy^45,46^. Further, it is important to mention that the employed assessment paradigm exhibits most hallmark features of human motor control in healthy controls during well-defined goal-directed tasks, including, for example, bell-shaped velocity profiles, safety margins, and aspects of movement-force coupling, thereby suggesting the generalizability of the obtained results to other behavioural tasks.

Interestingly, the observed results actually resemble those found previously at the muscular level, where a low-dimensional representation of few muscular components was found in healthy controls and also in mildly impaired post-stroke subject^13,17,18^. Also, these studies found impairment in the activation of these components^17,18^. Whereas the muscular components are clearly more directly related to neural control compared to our behavioural fPCs, one could still speculate that a relationship between the muscular and behavioural level exists. One way this might be implemented is that that the activation patterns of the muscular components are optimized to consistently produce specific kinematic and kinetic behavioural components, similar to the concept of a *fixed synergy*^19^. An alternative hypothesis would be that the kinematic and kinetic outputs are merely a by-product of task-related optimization and not directly controlled by the CNS^47^. Further research is needed to directly test these hypotheses by addressing the relationship between muscular and kinematic as well as kinetic components.

Further, it should be noted that decomposition algorithms are indeed able to capture underlying behavioural patterns, and that the obtained results are not merely a result of computational artifacts^13^. Compared to the information provided by typical kinematic metrics, which quantify impaired movement quality with a single value^6,12^, our applied decomposition algorithm provides complementary insights into two important aspects: First, even though it is intuitive that more fPCs are required to reconstruct temporal patterns composed by multiple sub-movements, the specific number of fPCs required to accurately reconstruct the signal allows to understand how simple (few fPCs) or complex (many fPCs) it can be to mathematically compose these altered behavioural patterns. Second, the visualization of the temporal evolution of the fPCs allows to put these behavioral patterns in a task-related context. These two aspects allowed to better understand the mechanisms underlying upper limb behaviours and their relevance for goal-directed tasks, which is not possible with typical kinematic metrics. This emphasizes that our approach focuses on better understanding the effects of stroke on motion composition whereas typical kinematic metrics rather aim to provide sensitive outcome measures that can be used in clinical studies.

### Potential clinical implications

Our observations can help inform future neurorehabilitation approaches. Specifically, the preservation of the fPCs in mild to moderately affected post-stroke individuals might suggest that these individuals do not need to relearn basic elements of motor control and instead need to optimize the recruitment and coordination of the remaining motor repertoire. In clinical terms, this could mean that chronic post-stroke therapy should focus, to a certain extent, on improving movement quality. While this has been already discussed in previous research^48,49^, we herein provide quantitative data that might motivate such an approach, which is rarely provided in literature. Further support for this suggestion is provided by recent clinical trials reporting significant gains in sensorimotor function following administration of high-dose and high-quality therapy that also considers movement quality^46,50^. However, dedicated strategies might be needed to relearn the underlying mechanics of controlling the reduction of grip forces, as we found alterations in the arrangement of the underlying fPCs. Also, post-stroke individuals showed a reduced ability to precisely control grip forces in a task-specific context. This indicates that it is important to practice meaningful daily life activities including object manipulations, instead of focusing on the pure production of forces without a task-related context.

### Limitations and future work

The results of this work should be interpreted with appropriate caution. While the number of tested participants is similar to related studies on patient populations^18^, the sample size does not allow capturing the full variability spectrum of mildly to moderately impaired stroke individuals. Similarly, the arrangement of muscular primitives has been shown to be altered in acute and sub-acute conditions or individuals with severe deficits^18^, which were not considered in this work. In the discussion of the results, we partially rely on visual inspection. Of note, we are aware that relying on this type of analysis could be a weakness of our work. However, in this paper, we did use visual analysis not as a primary outcome but as a way to present and discuss the results of the quantitative comparisons we reported. In the future, we will attempt a simultaneous decomposition of kinematic and kinetic data and further investigate computational approaches that allow to transform the information provided by the shape and variance of the fPCs into intuitively understandable metrics that can be used as clinical endpoints to longitudinally monitor sensorimotor recovery and evaluate the effect of interventions. In addition, we will focus on an in-depth comparison between the behavioural composition of goal-directed movements between the paretic and non-paretic side of post-stroke individuals, which was out of the scope of this work.

## Materials and methods

### Virtual Peg Insertion Test (VPIT)

The VPIT is a technology-based assessment that relies on a haptic end-effector (Phantom Omni or Geomagic Touch, 3D Systems, USA), a handle instrumented with piezoresistive sensors able to measure grasping forces, and a virtual reality environment visualizing a goal-directed object manipulation task^12,27^. In more detail, nine virtual pegs are aligned on a virtual pegboard and have to be transported into nine virtual holes by coordinating arm and hand movements as well as grasping forces. In order to lift a peg, the virtual cursor have to be spatially aligned with the virtual peg. Subsequently, a grasping force of at least 2 N has to be applied and maintained to transport the peg towards a hole. Upon entering the hole and releasing the grasping force, the virtual peg will be successfully inserted. To ease the perception of the virtual reality environment, the haptic end-effector provides a vertical force rendering the virtual pegboard. At the position of the holes, no vertical force is provided by the end-effector.

During task execution, kinematic (3D Cartesian translation) and kinetic (grasping force) data are recorded with a sampling rate of 1 kHz. Previously, a signal processing pipeline has been introduced to interpolate, filter, and segment these data (details in^12^): The first step linearly interpolates temporal gaps of at least 50 samples. The second step combines the 3D kinematic data into a single 1D distance trajectory by summing up their absolute first time-derivatives. Similarly, a 1D grasping force signal was generated by averaging across data from the three sensors. The third step involved the low-pass filtering of all sensor data using a zero-phase Butterworth filter (4th order, cut-off frequency 8 Hz). Subsequently, the distance and grasping force signals were temporally derived to obtain a velocity and force rate signal, respectively. Lastly, the data were segmented into different phases of interest that are expected to inform on varying aspects of sensorimotor control. This included the transport phase (gross movement from peg pickup until peg insertion) that involves the coordination of arm movements and grip forces. Further, the data was segmented in the return phase (gross movement from peg insertion until peg pickup). This led to nine and eight datapoints per task repetition for the transport and return phase, respectively. The start and end of these phases were defined upon passing and falling below a threshold (10% of maximum) in the velocity domain, respectively. To better isolate the contribution of grasping force control from arm movements, the force buildup and release phases were defined as the moments of rapid, maximal force production and reduction, respectively. Analogously to the transport/return phases, the buildup and release phases were defined through a threshold (10% of maximum) in the force rate (first time-derivative of grasping force) signal. The threshold was selected moving from the literature in the field^51^ and heuristically tuned for our purpose. Example data illustrating the phase segmentation are provided in previous work^12,52,53^.

### Functional principal component analysis

In order to characterize the behavioural data, a previously developed fPCA processing framework was applied to the velocity and force rate data of each movement phase^15,16^. First, to enable time-comparison of samples, we performed a time-warping of signals. Leveraging on the segmentation of different task phases, data associated with each phase were resampled to have the same number of time frames. Of note, with this process we lose the information on the average speed of the motion, which is not of interest in our analysis. However, all the other properties of the signal are preserved thanks to the constraints imposed to the time-warping, i.e. monotonicity, to preserve data integrity, and linear distortion of time. This enables to reach a consensus on the timing of different task repetitions with minimum loss of information.

Subsequently, fPCA was applied to describe the kinematic and kinetic data. fPCA is a statistical method that generalizes classical principal component analysis to time-series^54^. The core goal is the identification of a basis of R functions ${{\Sigma }} = { }\left[ {{\upxi }_{1} , {\upxi }_{2} , \ldots , {\upxi }_{{\text{R}}} } \right]$, which proper combination can reconstruct each entry of the dataset. Following the philosophy of classic PCA, basis elements are ordered such that the first functional principal component ${\upxi }_{1} \left( t \right)$ is the function for which the principal component scores $f_{i1} = \smallint {\upxi }_{1} \left( t \right) x_{i} \left( t \right)dt$ maximize $\mathop \sum \limits_{i} f_{i1}^{2}$ subject to $\smallint {\upxi }_{1}^{2} \left( t \right)dt = \left| {\xi_{1} } \right| = 1$; the second functional principal component $\xi_{2} \left( t \right)$ maximizes $\mathop \sum \limits_{i} f_{i2}^{2}$ subject to $\left| {\xi_{2} } \right| = 1$ and $\smallint {\upxi }_{1} \left( t \right) \xi_{2} \left( t \right)dt = 0$ (i.e. the second fPC is orthogonal to the first one) and so on. The practical implementation of fPCA, which bypass the solution of the optimization problem, is presented in previous work^15^. In a nutshell, for a given dataset, each fPC explains a certain percentage of variance, which is inversely proportional to the order of the fPC considered.

In the present work, we extracted the functional Principal Components on each body side separately. This choice was made to better identify the differences in motion control generated by the stroke.

### Participants and procedures

Thirty individuals post-stroke were enrolled at the University Hospital of Zurich (Zurich, Switzerland) and the cereneo Center for Neurology and Rehabilitation (Vitznau, Switzerland) as part of an observational study^12,28^. Details on subject enrolled in this study are reported in the Supplementary Material (see Table SM1). The study was approved by the Ethics Committee of ETH Zurich (ID 2016–02,075 and 2017–00,398) and all subjects gave written informed consent. The study was carried out in accordance with the guidelines of the Declaration of Helsinki. Included were only subjects of at least 18 years of age with chronic stroke. In addition, they had to have the ability to at least partially lift the arm against gravity and flex/extent the fingers. Reasons for exclusion from the study were concomitant diseases that influence upper limb function, severe sensory deficits, and severe cognitive impairment. The participants performed the FMA-UE and ARAT under instructions by the same trained healthcare practitioner. Clinical assessments were done specifically for this study and not as part of clinical routine. In addition, all subjects completed an initial familiarization with the VPIT followed by five repetitions (i.e., insertion of all nine pegs five times) of the test, starting with the most-affected body side. The interested reader is also invited to refer to^55^, in which we present and release this dataset in a larger framework of multiple multi-modal and multi-center data of healthy and stroke affected human upper limb movements. In order to provide normative reference performance values for the VPIT, an age-matched population of unaffected individuals were selected from a previously recorded normative database^12^.

### Data analysis

#### Statistical comparison of the shape of the behavioural time-series

The aim was to compare the shape of the behavioural time-series for each task phase between the control and stroke population. For this purpose, the time-series were visualized on a population level. Given that the task phases can be of different length for each subject and task repetition, we visualized the time-series after they were rescaled to the same length, as also required for the fPCA. The mean and standard deviation was used to aggregate the time-series across participants and enable a smooth visualization. Further, to quantify the similarity of the fPCs shape across subjects, their variability was evaluated using the average interquartile range (IQR) along the trajectory. To provide a quantitative description of the population-level differences in each time-series, we applied 1D statistical parametric mapping^56^. This methodology relies on random field theory to statistically compare time-series without requiring to repeatedly apply a windowing operation followed by a hypothesis test to the time-series, which has been shown to bias the statistical outcome^56^. With that, statistical parametric mapping allows to define the timepoints at which two sets of time-series are significantly different and describes these differences through a *t-* (normalized magnitude of difference) and *P*-value.

#### Statistical comparison of variance explained by fPCs

The amount of variance explained by the first six fPCs was compared within and between the control group (dominant and non-dominant side) and individuals post-stroke (non-paretic and paretic side). In addition, the number of fPCs required to cover 90% of the total variance (i.e., accurately reconstruct the input signal) were compared between the four subgroups. While it is intuitive that the amount of variance explained monotonically decreases with the number of fPCs, it is expected that the specific distribution of variance differs between healthy controls and post-stroke individuals. All statistical comparisons across subgroups were implemented using Kruskal–Wallis tests (i.e., non-parametric one-way ANOVAs) followed by Tukey–Kramer post-hoc tests.

#### Correlation analysis

Lastly, the variance explained by each fPC, signal, and task phase were compared to established clinical scales of describing sensorimotor impairments (FMA-UE) by calculating the Spearman correlation coefficients (ρ) for data collected with the most affected side. Data from the less affected side was not used due to the ceiling effect in the clinical scales that would bias the correlation analysis. The following intervals were used to interpret correlation coefficients: very high: ρ ≥ 0.9; high: 0.7 ≤ ρ < 0.9; moderate: 0.5 ≤ ρ < 0.7; low: 0.3 ≤ ρ < 0.5; very low: ρ < 0.3.

All data analysis procedures were implemented using MATLAB (MathWorks, MA, US) and scripts were written for a custom-made implementation of dynamic time-warping and fPCA.

## Supplementary Information

Supplementary Information.
