# Supplementary material for: A low-dimensional representation of arm movements and hand grip forces in post-stroke individuals
Source: Sci Rep. 2022 May 9;12:7601. doi: 10.1038/s41598-022-11806-4 (PMC9085765; doi:10.1038/s41598-022-11806-4)
Supplement: Supplementary file 1 — Supplementary Information. [file 41598_2022_11806_MOESM1_ESM.docx]

Supplementary Material for: A low-dimensional representation of arm movements and hand grip forces in post-stroke individuals

Christoph M. Kanzler^1,2†^, Giuseppe Averta^3,4†^, Anne Schwarz^5,6^, Jeremia P.O. Held^5,6^, Roger Gassert^1,2^, Antonio Bicchi^3,4^, Marco Santello^7^, Olivier Lambercy^1,2‡^, Matteo Bianchi^3‡^

^†‡^contributed equally

Corresponding authors: Christoph M. Kanzler, Giuseppe Averta

**Email:**, [relab.publications@hest.ethz.ch](mailto:relab.publications@hest.ethz.ch) , [g.averta3@gmail.com](mailto:g.averta3@gmail.com)

**Affiliations**

1 Rehabilitation Engineering Laboratory, Institute of Robotics and Intelligent Systems, Department of Health Sciences and Technology, ETH Zurich, Zurich, Switzerland.

2 Future Health Technologies, Singapore-ETH Centre, Campus for Research Excellence And Technological Enterprise (CREATE), Singapore.

3 Research Center “Enrico Piaggio” and Dipartimento di Ingegneria dell’Informazione, University of Pisa, Pisa, Italy.

4 Soft Robotics for Human Cooperation and Rehabilitation, Fondazione Istituto Italiano di Tecnologia, Genova, Italy.

5 Vascular Neurology and Neurorehabilitation, Department of Neurology, University Hospital and University of Zurich, Zurich, Switzerland.

6 Biomedical Signals and Systems (BSS), University of Twente, Enschede, The Netherlands.

7 School of Biological and Health Systems Engineering, Arizona State University, Tempe, AZ, USA.


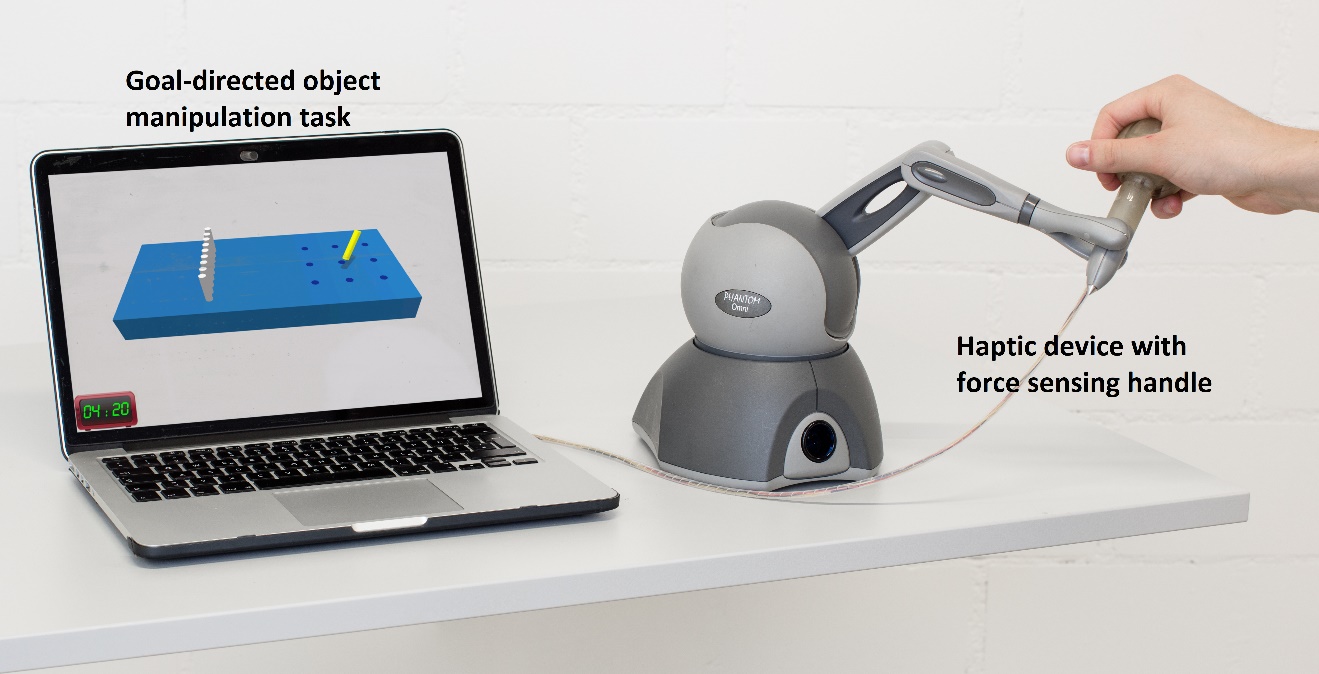


Figure SM1 The Virtual Peg Insertion Test, a technology-aided assessment of upper limb arm and hand function. The approach relies on a haptic end-effector device with a custom-made force sensing handle and goal-directed object manipulation task in a virtual reality environment rendered on a personal computer.


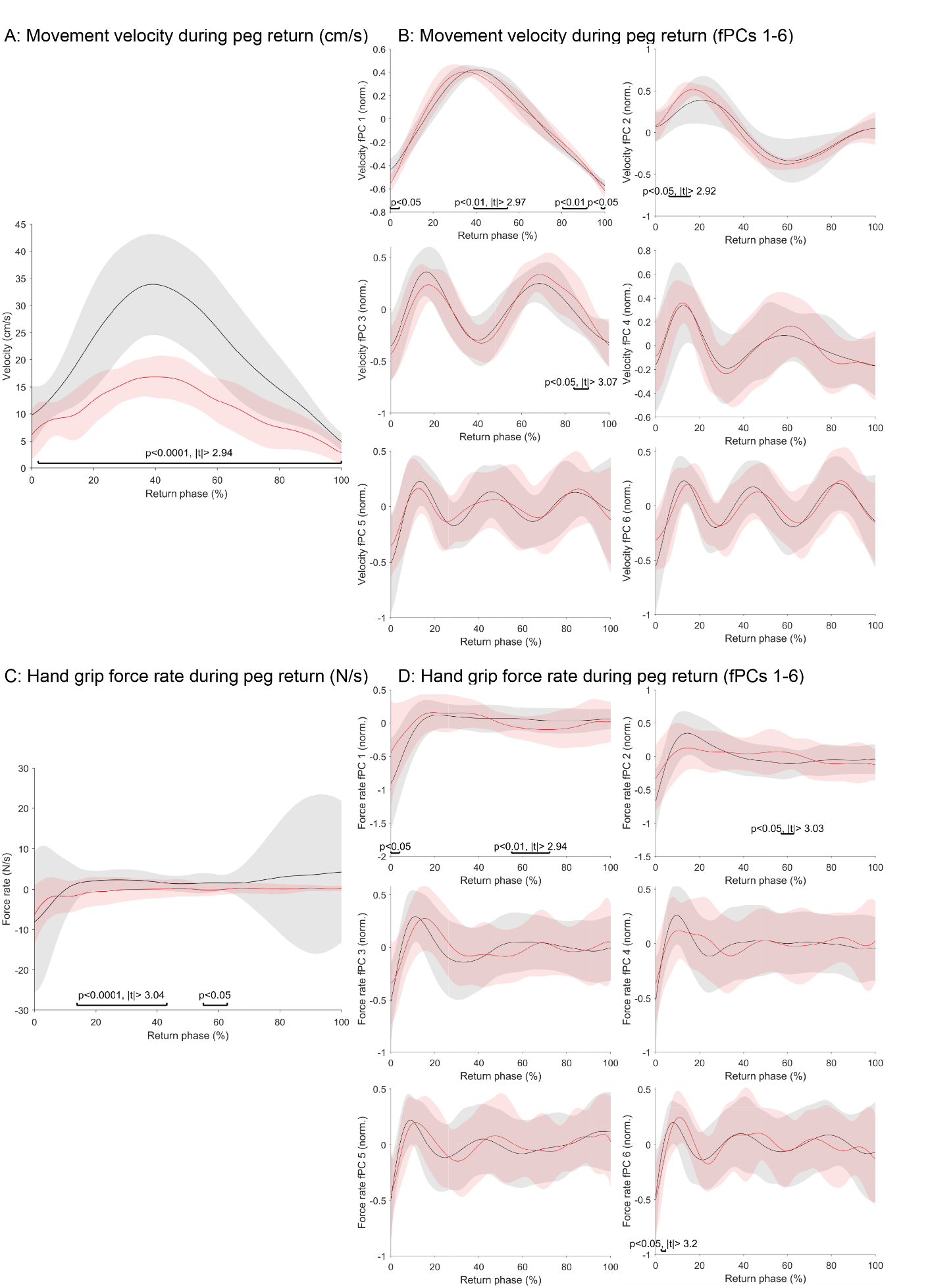


Figure SM2 Movement and grip force coordination during the return phase of the functional task. The preprocessed end-effector velocity (A) and grip force rate (C) signals were visualized for the paretic side of post-stroke subjects (red) and a healthy age-matched control population (gray). In addition, the shapes of the fPCs for the velocity (B) and the force rate (D) signals were visualized. The time-series of the stroke and control population were compared using statistical parametric mapping and the p- and t-values of significant periods annotated.

| **Participant ID** | **Age** | **Gender** | **Tested side** | **Paretic side** | **Chronicity** (weeks) | **FMA-UE**  (0-66) | **ARAT** (0-57) |
| --- | --- | --- | --- | --- | --- | --- | --- |
| 1 | 67 | Male | Right | Left | 113.0 | 66 | 57 |
| 2 | 55 | Male | Left | Left | 91.3 | 54 | 56 |
| 2 | 55 | Male | Right | Left | 91.3 | 66 | 57 |
| 3 | 55 | Male | Left | Right | 108.6 | 65 | 57 |
| 3 | 55 | Male | Right | Right | 108.6 | 49 | 55 |
| 4 | 52 | Male | Left | Left | 147.7 | 55 | 52 |
| 4 | 52 | Male | Right | Left | 147.7 | 65 | 57 |
| 5 | 73 | Male | Left | Right | 48.1 | 62 | - |
| 6 | 69 | Female | Right | Left | 46.3 | 61 | 57 |
| 7 | 67 | Male | Left | Left | 130.4 | 50 | - |
| 7 | 67 | Male | Right | Left | 130.4 | 66 | - |
| 8 | 40 | Female | Left | Right | 41.7 | 56 | 45 |
| 8 | 40 | Female | Right | Right | 41.7 | 49 | 49 |
| 9 | 71 | Male | Left | Left | 242.7 | 40 | 35 |
| 9 | 71 | Male | Right | Left | 242.7 | 65 | 57 |
| 10 | 59 | Female | Left | Left | 235.1 | 50 | 47 |
| 10 | 59 | Female | Right | Left | 235.1 | 66 | 57 |
| 11 | 88 | Female | Left | Left | 89.1 | 37 | 39 |
| 11 | 88 | Female | Right | Left | 89.1 | 63 | - |
| 12 | 69 | Female | Left | Right | 31.6 | 63 | 57 |
| 12 | 69 | Female | Right | Right | 31.6 | 44 | 39 |
| 13 | 59 | Female | Left | Right | 104.9 | 66 | 57 |
| 13 | 59 | Female | Right | Right | 104.9 | 57 | 56 |
| 14 | 50 | Female | Right | Left | 260.7 | 64 | - |
| 15 | 61 | Male | Left | Right | 469.7 | 66 | 56 |
| 15 | 61 | Male | Right | Right | 469.7 | 38 | 42 |
| 16 | 59 | Male | Left | Left | 88.3 | 46 | 40 |
| 16 | 59 | Male | Right | Left | 88.3 | 63 | 57 |
| 17 | 69 | Male | Left | Left | 27.7 | 53 | 51 |
| 17 | 69 | Male | Right | Left | 27.7 | 63 | 56 |
| 18 | 55 | Male | Left | Left | 78.2 | 59 | 57 |
| 18 | 55 | Male | Right | Left | 78.2 | 66 | 57 |
| 19 | 42 | Male | Left | Left | 26.1 | 39 | 30 |
| 19 | 42 | Male | Right | Left | 26.1 | 65 | 57 |
| 20 | 51 | Female | Left | Right | 52.1 | 66 | 57 |
| 20 | 51 | Female | Right | Right | 52.1 | 61 | 57 |
| 21 | 58 | Male | Left | Right | 26.1 | 62 | 57 |
| 21 | 58 | Male | Right | Right | 26.1 | 42 | 53 |
| 22 | 46 | Male | Left | Left | 56.5 | 57 | 42 |
| 22 | 46 | Male | Right | Left | 56.5 | 66 | 57 |
| 23 | 76 | Male | Left | Right | 147.7 | 66 | 55 |
| 23 | 76 | Male | Right | Right | 147.7 | 60 | 54 |
| 24 | 53 | Female | Left | Right | 160.8 | 66 | 57 |
| 24 | 53 | Female | Right | Right | 160.8 | 58 | 55 |
| 25 | 62 | Male | Left | Right | 790.8 | 66 | 57 |
| 25 | 62 | Male | Right | Right | 790.8 | 34 | 33 |
| 26 | 62 | Male | Left | Right | 56.5 | 64 | 57 |
| 26 | 62 | Male | Right | Right | 56.5 | 46 | 43 |
| 27 | 69 | Male | Left | Right | 52.1 | 60 | 54 |
| 27 | 69 | Male | Right | Right | 52.1 | 32 | 34 |

Table SM1 Detailed demographics for the post-stroke population. FMA-UE: Fugl-Meyer Assessment Upper-Extremity. ARAT: Action Research Arm Test. For the correlation analysis with the clinical scores, only subjects that had both an ARAT and FMA-UE score was used. All post-stroke individuals were right-hand dominant, except participant 9.

Table SM2 Correlations between the variance explained per fPC and the Action Research Arm Test (ARAT). The Spearman correlation analysis was performed for the paretic side of post-stroke subjects. Bold indicates significant entries. *p<0.05. **p<0.001.

| **Signal & task phase** | **Correlations of fPCs with ARAT** | | | | | |
| --- | --- | --- | --- | --- | --- | --- |
|  | fPC1 | fPC2 | fPC3 | fPC4 | fPC5 | fPC6 |
| Velocity transport | **0.40*** | -0.25 | **-0.40*** | **-0.46*** | **-0.46*** | **-0.46*** |
| Force rate transport | **0.52**** | **-0.43*** | **-0.54**** | **-0.52**** | **-0.53**** | **-0.51**** |
| Velocity return | **0.47*** | -0.25 | **-0.58**** | **-0.48**** | **-0.47*** | **-0.45*** |
| Force rate return | 0.17 | 0.10 | -0.21 | -0.33 | -0.22 | -0.24 |
| Force rate buildup | 0.18 | 0.14 | -0.33 | -0.16 | -0.12 | -0.21 |
| Force rate release | **0.40*** | **-0.41*** | **-0.36*** | 0.27 | **-0.32*** | **-0.39*** |
